# Thrombotic risk assessment in antiphospholipid syndrome: do noncriteria antibodies contribute?

**DOI:** 10.55730/1300-0144.5671

**Published:** 2023-09-13

**Authors:** Ömer ULUDAĞ, Suzan ÇINAR, Thomas MCDONNELL, Erhan ÇENE, Yasemin YALÇINKAYA, Ahmet GÜL, Murat İNANÇ, Bahar ARTIM ESEN

**Affiliations:** 1 Division of Rheumatology, Department of Internal Medicine, İstanbul Faculty of Medicine, İstanbul University, İstanbul, Turkiye; 2 Department of Immunology, Aziz Sancar Institute of Experimental Medicine, İstanbul University, İstanbul, Turkiye; 3 Centre for Rheumatology Research, Division of Medicine, University College London, London, United Kingdom; 4 Department of Statistics, Faculty of Arts and Science, Yıldız Technical University, İstanbul, Turkiye

**Keywords:** Antiphospholipid syndrome, global antiphospholipid syndrome score, thrombotic risk, non-criteria antiphospholipid antibodies

## Abstract

**Background/aim:**

In this cross-sectional study, it was aimed to test the predictive value of noncriteria antiphospholipid antibodies (aPL) in addition to the global antiphospholipid syndrome score (GAPSS) in predicting vascular thrombosis (VT) in a cohort of patients with APS and aPL (+) systemic lupus erythematosus (SLE).

**Material and methods:**

This study included 50 patients with primary APS, 68 with SLE/APS, and 52 with aPL (+) SLE who were classified according to VT as VT ± pregnancy morbidity (PM), PM only or aPL (+) SLE. Antiphospholipid serology consisting of lupus anticoagulant (LA), anticardiolipin (aCL) immunoglobulin G (IgG)/IgM/IgA, antibeta2 glycoprotein I (aβ2GPI) IgG/IgM/IgA, antiphosphatidylserine/prothrombin (aPS/PT) IgG/IgM and antidomain-I (aDI) IgG was determined for each patient. The GAPSS and adjusted GAPSS (aGAPSS) were calculated for each patient, as previously defined. Logistic regression analysis was carried out with thrombosis as the dependent variable and high GAPSS, aCL IgA, aβ2GPI IgA, and aDI IgG as independent variables.

**Results:**

The mean GAPSS and aGAPSS of the study population were 11.6 ± 4.4 and 9.6 ± 3.8. Both the VT ± PM APS (n = 105) and PM only APS (n = 13) groups had significantly higher GAPSS and aGAPSS values compared to the aPL (+) SLE (n = 52) group. The patients with recurrent thrombosis had higher aGAPSS but not GAPSS than those with a single thrombotic event. The computed area under the receiver operating characteristic curve demonstrated that a GAPSS ≥13 and aGAPSS ≥10 had the best predictive values for thrombosis. Logistic regression analysis including a GAPSS ≥13, aCL IgA, aβ2GPI IgA, and aDI IgG showed that none of the factors other than a GAPSS ≥13 could predict thrombosis.

**Conclusion:**

Both the GAPSS and aGAPSS successfully predict the thrombotic risk in aPL (+) patients and aCL IgA, aβ2GPI IgA, and aDI IgG do not contribute to high a GAPSS or aGAPSS.

## 1. Introduction

Antiphospholipid syndrome (APS) is an autoimmune disease characterized by the persistent presence of antiphospholipid antibodies (aPL) in association with arterial, venous, or small vessel thrombosis and/or pregnancy morbidity (PM) [1]. Thrombotic events are the leading cause of mortality in patients with APS [2]. Therefore, identifying patients with high risk of thrombosis is essential.

Lupus anticoagulant (LA) positivity and triple aPL [simultaneous presence of LA, anticardiolipin (aCL) and antibeta2 glycoprotein I (aβ2GPI)] positivity are accepted as high-risk serological profiles for thrombosis [3,4]. In addition, immunoglobulin A (IgA) isotypes of aCL and aβ2GPI and antiphosphatidylserine/prothrombin (aPS/PT) antibodies, which are so-called noncriteria aPLs, have been found to be associated with both vascular thrombosis (VT) and PM [5,6]. Antidomain-I (aDI) antibodies against the cryptogenic epitope of β2GPI, are also strongly associated with thrombotic and obstetric events in APS [7].

Two scoring systems have been developed to assess the thrombotic risk in APS. While the antiphospholipid score (aPL-S) is a scoring based solely on the serological profile (three different clotting assays to detect LA and IgG/IgM isotypes of aCL, β2GPI and aPS/PT), the global antiphospholipid syndrome score (GAPSS) includes conventional cardiovascular risk factors (arterial hypertension and hyperlipidemia) in addition to the serological profile (LA and IgG/IgM isotypes of aCL, β2GPI, and aPS/PT) [8,9]. Since aPS/PT is not included in the APS classification criteria and is not routinely tested in many centers, the GAPSS has been simplified as the adjusted GAPSS (aGAPSS) by excluding aPS/PT [10]. The validation of the GAPSS and/or aGAPSS in different cohorts of patients with primary APS (PAPS) and systemic lupus erythematosus (SLE) ± APS verified the association of higher scores with a higher risk of thrombosis [10–15]. Furthermore, the GAPSS/aGAPSS scores were even higher in APS patients with recurrent thrombosis compared to those with a single thrombotic event [15–17].

Whether the inclusion of noncriteria aPLs increases the predictive value of existing scores remains an unanswered question. Herein, it was aimed to test the predictive value of noncriteria aPL in addition to the GAPSS in predicting VT in a cohort of patients with APS and aPL (+) SLE.

## 2. Methods

### 2.1. Patients

This single center, cross-sectional study included 118 consecutive patients with APS (50 PAPS and 68 SLE/APS) and 52 patients with aPL (+) SLE (at least one aPL positivity but no thrombotic or obstetric event) who were followed up at the weekly SLE/APS outpatient clinic by a standard protocol between 1982 and 2020. All of the patients with APS and SLE fulfilled the Sydney and Systemic Lupus International Collaborating Clinics (SLICC) classification criteria, respectively [1,18]. All of the aPL (+) SLE patients met at least one laboratory criterion of the Sydney classification criteria. The study was conducted in accordance with the declaration of Helsinki and was approved by Istanbul Faculty of Medicine Clinical Research Ethics Committee (approval number: 2018/1679). Written informed consent to participate and publish the results was obtained from all patients.

Data regarding demographic, clinical and laboratory characteristics of the patients were retrieved retrospectively from the database. Disease duration was defined as the time from the diagnosis of APS to the time of the last visit for the patients with APS and as the time from the diagnosis of SLE to the time of last visit for the patients with aPL (+) SLE. VT was documented using an appropriate imaging method (Doppler ultrasonography, computed tomography, magnetic resonance imaging, or ventilation-perfusion scintigraphy). Livedo reticularis, thrombocytopenia, heart valve disease and APS nephropathy (APSN) were included as extra criteria manifestations. Livedo reticularis was assessed by physical examination. Thrombocytopenia was defined as a platelet count of <100 × 10^9^/mm^3^ in at least two examinations and was confirmed by a peripheral blood smear. Heart valve disease was confirmed with echocardiography and APSN was documented with kidney biopsy [1].

Data regarding cardiovascular risk factors (arterial hypertension, hyperlipidemia, diabetes, and obesity) were collected and revised. Only those antecedent to thrombotic or obstetric events were taken into consideration. Arterial hypertension was defined as systolic blood pressure (SBP) values ≥140 mmHg and/or diastolic blood pressure (DBP) values ≥90 mmHg at least in 2 occasions or being under treatment with antihypertensive drugs [19]. Hyperlipidemia was defined as low-density lipoprotein cholesterol ≥160 mg/dL and/or triglyceride ≥175 mg/dL in at least two measurements or the use of statin therapy [20]. Patients who met at least one of the following criteria were diagnosed as diabetes mellitus: the fasting plasma glucose (PG) ≥126 mg/dL, PG ≥200 mg/dL after 2-h of a 75-g oral glucose tolerance test, a random PG ≥ 200 mg/dL in a patient with hyperglycemic symptoms, hemoglobin A1c ≥6.5%, and being under treatment with insulin or oral antidiabetic drugs [21]. Patients with a body mass index (BMI) ≥30 were considered as obese.

### 2.2. aPL detection

The aPL profile included LA, aCL, aβ2GPI, aPS/PT, and aDI antibodies. LA was measured by aPTT and diluted Russell viper venom time assays at the hematology laboratory according to the guidelines of the International Society on Thrombosis and Haemostasis [22]. The aCL IgG/IgM/IgA, aβ2GPI IgG/IgM/IgA, and aPS/PT IgG/IgM antibodies were detected by enzyme-linked immunosorbent assay (ELISA) (QUANTA Lite ELISA assays; Inova Diagnostics, San Diego, CA, USA) and the positivity threshold was accepted as >40 phospholipid units for aCL IgG/IgM/IgA (GPLU/MPLU/APLU, respectively); as >20 units for aβ2GPI IgG/IgM/IgA (GBU/MBU/ABU, respectively); and as >30 units for aPS/PT IgG/IgM. aDI IgG antibodies were measured by in-house ELISA tests in the laboratory of Rheumatology Research Unit, University College London as previously described [23]. The positivity was defined as titers >99th percentile and cut-offs for positivity were determined to as 14 anti-DI units.

GAPSS and aGAPSS were calculated as previously defined by adding corresponding points to the risk factors: 3 for hyperlipidemia, 1 for arterial hypertension, 5 for aCL IgG/IgM, 4 for aβ2GPI IgG/IgM, 4 for LA, and 3 for aPS/PT IgG/IgM [8].

### 2.3. Statistical analysis

Continuous variables were expressed as the mean ± standard deviation (SD) if they were normally distributed, and otherwise as the median (interquartile range). Categorical variables were expressed as the number (%). Comparisons of the continuous variables between two groups were performed using the t test or the Mann–Whitney U test; ANOVA or Kruskal–Wallis tests were used when comparing three or more groups. A two-sided p-value < 0.05 was considered statistically significant.

The diagnostic powers of the GAPSS and aGAPSS for thrombosis development were assessed by receiver operating characteristic (ROC) curve analysis. The sensitivity, specificity, positive predictive value (PPV), and negative predictive value (NPV) of the different GAPSS and aGAPSS cut-off values were determined. Logistic regression analysis was carried out with thrombosis as the dependent variable and high GAPSS, aCL IgA, aβ2GPI IgA, and aDI IgG as independent variables.

## 3. Results

Included in this analysis were 50 patients with PAPS, 68 with SLE/APS, and 52 with aPL (+) SLE. Among the patients with APS (n = 118), 71 (60.2%) had only VT, 13 (11%) had only PM, and 34 (28.8%) had both. Of 105 patients with thrombosis, 50 (47.6%) had only arterial, 30 (28.6%) had only venous, 22 (21%) had both arterial and venous thrombosis, while 3 (2.9%) had small vessel thrombosis. Of the patients with thrombosis, 43 (40.9%) had recurrence. The most common thrombotic events were ischemic stroke (n = 55, 32.4%), deep vein thrombosis (n = 40, 23.5%) and pulmonary embolism (n = 14, 8.2%). Late pregnancy losses (n = 32, 68%) ranked first among PM (n = 47) presentations followed by early losses (n = 11, 23.4%), preeclampsia/eclampsia (n = 11, 23.4%), and premature birth (n = 7, 14.9%).

The most common cardiovascular risk factor was hypertension (n = 79, 46.5%) which was followed by hyperlipidemia (n = 63, 37.1%), obesity (n = 53, 31.2%), and smoking (n = 45, 26.5%), respectively. Demographic and clinical characteristics of the patients with PAPS, SLE/APS, and aPL (+) SLE are presented in Table 1.

Among the aPLs included in the classification criteria, LA positivity was the most frequent (75.3%), which was followed by aCL IgG (50%), aβ2GPI IgG (38.2%), aCL IgM (26.5%), and aβ2GPI IgM (25.3%). Frequencies of LA, aCL IgG, aβ2GPI IgG, aβ2GPI IgM, and aPS/PT IgM were higher in the patients with APS (PAPS and SLE/APS) compared to those with aPL (+) SLE (81.4% vs. 61.5%, p = 0.006; 61.9% vs. 23.1%, p < 0.001; 46.6% vs. 19.2%, p < 0.001; 31.4% vs. 11.5%, p = 0.006; and 55.9% vs. 38.5%, p = 0.036, respectively). Comparison of the criteria and noncriteria aPL frequencies between the VT ± PM APS, PM only APS, and aPL (+) SLE groups is shown in Table 2.

The mean GAPSS and aGAPSS of the study population were 11.6 ± 4.4 and 9.6 ± 3.8. Both the VT ± PM APS and PM only APS groups had significantly higher GAPSS and aGAPSS values compared to the aPL (+) SLE group (GAPSS 12.9 ± 4.4 vs. 8.9 ± 3.4, p < 0.001 and 11.9 ± 3.6 vs. 8.9 ± 3.4, p = 0.006, respectively; aGAPSS 10.7 ± 3.9 vs. 7.2 ± 2.9, p < 0.001 and 10.1 ± 2.9 vs. 7.2 ± 2.9, p = 0.002, respectively). Despite higher GAPSS and aGAPSS scores in the VT ± PM APS group, the difference was not statistically significant compared to the PM only APS group (p = 0.42 and p = 0.51 respectively). The patients with recurrent thrombosis had a higher aGAPSS than those with a single thrombotic event (aGAPSS 11.1 ± 4.2 vs. 9.2 ± 3.8, p = 0.008). Comparison of the GAPSS between the patients with recurrent thrombosis to those with a single thrombotic event did not reveal any statistically significant differences despite an inclination for a higher score in the patients with recurrent thrombosis (GAPSS 13.4 ± 4.7 vs. 12.9 ± 4.3, p = 0.548). In addition, patients with at least one extra criteria manifestation (n = 92) had a higher GAPSS and aGAPSS than those who had none (n = 78) (GAPSS 12.3 ± 4.5 vs. 10.9 ± 4.4, p = 0.038; aGAPSS 10.1 ± 3.8 vs. 8.9 ± 3.8, p = 0.038).

The diagnostic powers of the GAPSS and aGAPSS for thrombosis development were assessed by ROC curve analysis (Figure). The area under a ROC curve (AUC) for the GAPSS was 0.727 (95% CI: 0.651–0.803, p < 0.001) and AUC for aGAPSS was 0.715 (95% CI: 0.638–0.792, p < 0.001). Sensitivity, specificity, PPV, and NPV of the different GAPSS and aGAPSS cut-off values are shown in Table 3. The calculated AUC demonstrated that a GAPSS ≥13 and aGAPSS ≥10 had the best predictive values for thrombosis.

Separate logistic regression analyses on predicting VT using a GAPSS ≥13 and aGAPSS ≥10 with noncriteria aPL antibodies (aCL IgA, aβ2GPI IgA, and aDI IgG) showed that none of the factors other than a GAPSS ≥13 and aGAPSS ≥10 could predict VT (Table 4).

## 4. Discussion

Since VT is the leading cause of mortality in patients with APS, thrombotic risk stratification is crucial [2]. Previous studies that assessed the thrombotic risks of different aPL profiles had variable results [3,4,24–26]. Especially LA positivity and triple APL positivity are associated with high thrombotic risk. The GAPSS, and its simplified version, the aGAPSS, which were developed to quantify thrombotic risk, include aPL serology along with conventional cardiovascular risk factors (hypertension and hyperlipidemia) [8,10]. Both scores have been validated in different retrospective patient cohorts with APS (± SLE) [10–15]. Different GAPSS (aGAPSS) cut-off values have been reported from many centers as a reflection of discrepancies in serological and clinical characteristics of patient populations. Therefore, although high GAPSS values are associated with an increased risk of thrombosis, it has been suggested that each center should determine its own cut-off value [13,14]. In the current study, the cut-off GAPSS and aGAPSS values for thrombosis were determined as 13 and 10, respectively. The reason for the slightly higher cut-off values in the current cohort compared to previous ones was interpreted as the higher frequencies of aPL positivity, hypertension, and hyperlipidemia in the present patient population compared to previous cohorts [8,10,12–14]. In addition, the presence of patients with various autoimmune diseases other than APS and SLE in these cohorts might also have contributed to lower scores. Previous studies have shown higher GAPSS and aGAPSS values in patients with recurrent thrombosis [17,27]. Similarly, in the present study, patients with recurrent thrombosis had higher aGAPSS but not GAPSS values than those with a single thrombotic event. Failure to demonstrate this difference in the GAPSS may reflect that aPS/PT antibodies are insufficient to predict recurrent thrombosis in thrombotic APS. Previous studies have reported that antiprothrombin (aPT) and aPS/PT antibodies are associated with increased risk of thrombosis, and this relationship is stronger with aPS/PT antibodies [28]. However, since these studies mainly included patients with SLE, they did not reveal the association of aPS/PT with the recurrent thrombosis in thrombotic APS. The results herein may imply that the aGAPSS, in addition to its convenience in daily practice, may be a better predictor for recurrent thrombosis than GAPSS in patients with thrombotic APS.

Although the IgA isotypes of aCL and aβ2GPI are included in the SLE classification criteria, they are not included in the APS classification criteria and are not widely used in daily practice [1,18]. However, it is well known that some seronegative APS cases have only aCL IgA or aβ2GPI IgA positivity, and there is evidence showing that especially aβ2GPI IgA is associated with thrombotic and obstetric APS [5,23,29]. In a prospective study including 821 patients with SLE, LA was shown to be the best predictor of thrombosis and only aβ2GPI IgA further increased the thrombotic risk in the LA positives [26]. Recently, the role of aDI, which was developed against the antigenically dominant region of β2GPI, has been investigated in the diagnosis and thrombotic risk stratification of APS. In an international multicenter study including 477 patients with aβ2GPI positivity (364 of whom met the APS classification criteria), aDI IgG antibodies were detected in 243 (55%) patients and found to be strongly associated with VT and PM [7]. In contrast, De Craemer et al. showed that aDI IgG positivity was associated with thrombotic APS but did not have an additional contribution to the diagnosis of APS and to the assessment of thrombotic risk in patients with aβ2GPI IgG positivity [30]. In a cross-sectional study including 111 patients with APS, 119 patients with SLE, and 200 healthy controls, it was shown that the presence of aDI IgG/IgM/IgA in patients with aCL/aβ2GPI positivity increased the hazard ratio for APS diagnosis by 3–5-fold [23]. In addition, IgG isotypes of aCL, aβ2GPI, and aDI, and the IgA isotype of aDI were found to be associated with VT but not PM [23]. In the current study, compared to that cohort, the frequency of aCL IgA was lower in both the APS and aPL (+) SLE groups, while the frequency of aβ2GPI IgA was similar in the APS group, but much higher in the aPL (+) SLE group. In addition, compared to that cohort, the frequency of aDI IgG was lower in the APS group, while was higher in the aPL (+) SLE groups. Since the aβ2GPI IgA and aDI IgG frequencies were similar in the thrombotic APS and aPL (+) SLE groups and the aCL IgA frequency was low in both, logistic regression analysis showed that none of these noncriteria antibodies make any contribution to GAPSS in thrombotic risk prediction.

The present study had some limitations. Its cross-sectional design may preclude the detection of changes in the risk scores over time, as aPL serology may change over time, particularly in association with SLE disease activity and immunosuppressive treatment. A prospective study with consecutive aPL detections and SLE disease activity assessments would allow a stronger clinical interpretation to be made. Although only the cardiovascular risk factors antecedent to thrombotic/obstetric events were taken into consideration, the duration and treatment of these risk factors may determine their effects on thrombosis. While the current study included a reasonable number of patients for a single-center study, the limited number of patients may have affected the subgroup comparisons, as in the case of the GAPSS comparison between patients with recurrent thrombosis and a single thrombotic event. Similarly, the limited number of APS patients with only PM in the present study made it difficult to make an interpretation about this group. Finally, since criteria aPL (+) patients we included, it was not possible to draw conclusions about the comparative risk of thrombosis associated with criteria vs. noncriteria aPL.

In conclusion, both the GAPSS and aGAPSS successfully predict the risk of thrombosis in aPL (+) patients. The aGAPSS may be superior to the GAPSS due to its ease of application in clinical practice and better ability to predict the risk of recurrent thrombosis in thrombotic APS. When evaluating the risk of thrombosis, each center should interpret the score according to its own cut-off value. IgA isotypes of aCL and aβ2GPI, and aDI IgG do not contribute to a high GAPSS in determining the risk of thrombosis. A possible explanation for this may be the transient positivity of these antibodies associated with disease activity in SLE. Prospective studies with consecutive antibody measurements are needed to rule out this possibility.

## Figures and Tables

**Figure:**
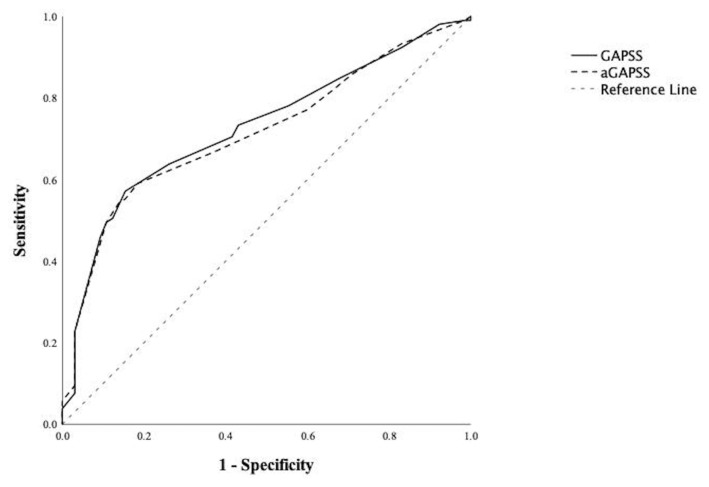
ROC curve analysis of the GAPSS and aGAPSS for thrombosis development.

**Table 1:** Demographic and clinical characteristics of the patients with PAPS, SLE/APS, and aPL (+) SLE.

	PAPS (n = 50)	SLE/APS (n = 68)	aPL (+) SLE (n = 52)	p-value
Female, n (%)	41 (82)	58 (85.3)	43 (82.7)	0.876
Age (years), mean (± SD)	43.4 (11.3)	40.6 (10)	41.9 (12.8)	0.407
Age at diagnosis (years), median (IQR)	32 (26–41)	31.5 (23.5–38)	29.5 (23.5–37)	0.359
Duration of disease (years), median (IQR)	6.6 (2.9–14.5)	8.1 (3.3–12.9)	12.9 (4.5–18.4)	0.076
Vascular thrombosis, n (%)	43 (86)	62 (91.2)	-	0.276
Arterial	29 (58)	43 (63.2)	-	0.411
Venous	23 (46)	29 (42.6)	-	0.430
Recurrent thrombosis	18 (36)	25 (36.8)	-	0.544
Pregnancy morbidity, n (%)	21 (42)	26 (38.2)	-	0.411
Miscarriages (≥3)	6 (12)	5 (7.4)	-	0.203
Fetal death	15 (30)	17 (25)	-	0.345
Pre-eclampsia/eclampsia	3 (6)	8 (11.8)	-	0.231
Premature birth	1 (2)	6 (8.8)	-	0.122
Extra-criteria manifestations, n (%)				
Livedo reticularis	2 (4)	13 (19.1)	10 (19.2)	0.039
Thrombocytopenia	9 (18)	28 (41.2)	15 (28.8)	0.025
Heart valve disease	13 (26)	26 (38.2)	7 (13.5)	0.010
APS nephropathy	3 (6)	8 (11.8)	0 (0)	0.034
Cardiovascular risk factors, n (%)				
Arterial hypertension	20 (40)	41 (60.3)	18 (34.6)	0.011
Hyperlipidemia	20 (40)	23 (33.8)	20 (38.5)	0.766
Diabetes mellitus	4 (8)	5 (7.4)	5 (9.6)	0.903
Obesity	21 (42)	19 (27.9)	13 (25)	0.136

APS: antiphospholipid syndrome, PAPS: primary APS, SLE: systemic lupus erythematosus, aPL: antiphospholipid antibody, IQR: interquartile range.

**Table 2:** Comparison of the aPL frequencies between VT ± PM APS, PM only APS, and aPL (+) SLE.

	All (n = 170)	VT ± PM APS (n = 105)	PM only APS (n = 13)	aPL (+) SLE (n = 52)	p-value
Triple aPL, n (%)	47 (27.6)	41 (39)	4 (30.8)	2 (3.8)	<0.001
LA, n (%)	128 (75.3)	89 (84.8)	7 (53.8)	32 (61.5)	0.001
aCL IgG, n (%)	85 (50)	63 (60)	10 (76.9)	12 (23.1)	<0.001
aCL IgM, n (%)	45 (26.5)	28 (26.7)	5 (38.5)	12 (23.1)	0.530
aCL IgA, n (%)	13 (7.6)	10 (9.5)	0 (0)	3 (5.8)	0.395
aβ2GPI IgG, n (%)	65 (38.2)	47 (44.8)	8 (61.5)	10 (19.2)	0.002
aβ2GPI IgM, n (%)	43 (25.3)	32 (30.5)	5 (38.5)	6 (11.5)	0.019
aβ2GPI IgA, n (%)	84 (49.4)	52 (49.5)	4 (30.8)	28 (53.8)	0.330
aPS/PT IgG, n (%)	85 (50)	58 (55.2)	4 (30.8)	23 (44.2)	0.152
aPS/PT IgM, n (%)	86 (50.6)	58 (55,2)	8 (61.5)	20 (38.5)	0.101
aDI IgG, n (%)	35 (20.6)	22 (21.0)	2 (15.4)	11 (21.2)	0.890

VT: vascular thrombosis, PM: pregnancy morbidity, LA: lupus anticoagulant, aCL: anticardiolipin, aβ2GPI: antibeta2-glycoprotein-I, aPS/PT: antiphosphatidylserine/prothrombin, aDI: antidomain-I.

**Table 3:** Diagnostic accuracy for thrombosis, including sensitivity, specificity, PPV, and NPV of the different cut-off values of the GAPSS and aGAPSS.

	Sensitivity	Specificity	PPV	NPV
GAPSS cut-off				
12	0.638	0.738	0.798	0.558
13	0.571	0.846	0.857	0.550
14	0.505	0.877	0.869	0.523
aGAPSS cut-off				
9	0.667	0.631	0.745	0.539
10	0.590	0.815	0.838	0.552
11	0.552	0.846	0.853	0.539

GAPSS: global APS score, aGAPSS: adjusted GAPSS, PPV: positive predictive value, NPV: negative predictive value.

**Table 4:** Logistic regression analysis for VT.

	OR (95% CI)	p-value		OR (95% CI)	p-value
GAPSS ≥13	7.76 (3.50–17.20)	<0.001	aGAPSS ≥10	6.63 (3.13–14.06)	<0.001
aCL IgA	2.25 (0.50–10.03)	0.288	aCL IgA	2.38 (0.54–10.53)	0.251
aβ2GPI IgA	0.67 (0.33–1.38)	0.276	aβ2GPI IgA	0.72 (0.35–1.47)	0.371
aDI IgG	1.01 (0.42–2.48)	0.982	aDI IgG	0.90 (0.37–2.19)	0.823
Cox–Snell R square = 0.177, Nagelkerke R square = 0.241, model significance <0.001			Cox–Snell R square = 0.164, Nagelkerke R square = 0.223, model significance <0.001		

OR: odds ratio, CI: confidence interval.

## References

[1] Miyakis S, Lockshin MD, Atsumi T, Branch DW, Brey RL (2006). International consensus statement on an update of the classification criteria for definite antiphospholipid syndrome (APS). Journal of Thrombosis and Haemostasis.

[2] Cervera R, Serrano R, Pons-Estel GJ, Ceberio-Hualde L, Shoenfeld Y (2015). Morbidity and mortality in the antiphospholipid syndrome during a 10-year period: a multicentre prospective study of 1000 patients. Annals of the Rheumatic Diseases.

[3] Ruffatti A, Del Ross T, Ciprian M, Bertero MT, Sciascia S (2011). Risk factors for a first thrombotic event in antiphospholipid antibody carriers: a prospective multicentre follow-up study. Annals of the Rheumatic Diseases.

[4] Pengo V, Biasiolo A, Pegoraro C, Cucchini U, Noventa F (2005). Antibody profiles for the diagnosis of antiphospholipid syndrome. Thrombosis and Haemostasis.

[5] Žigon P, Podovšovnik A, Ambrožič A, Tomšič M, Hočevar A (2019). Added value of non-criteria antiphospholipid antibodies for antiphospholipid syndrome: lessons learned from year-long routine measurements. Clinical Rheumatology.

[6] Shi H, Zheng H, Yin YF, Hu QY, Teng JL (2018). Antiphosphatidylserine/prothrombin antibodies (aPS/PT) as potential diagnostic markers and risk predictors of venous thrombosis and obstetric complications in antiphospholipid syndrome. Clinical Chemistry and Laboratory Medicine.

[7] de Laat B, Pengo V, Pabinger I, Musial J, Voskuyl AE (2009). The association between circulating antibodies against domain I of beta2-glycoprotein I and thrombosis: an international multicenter study. Journal of Thrombosis and Haemostasis.

[8] Sciascia S, Sanna G, Murru V, Roccatello D, Khamashta MA (2013). GAPSS: the global anti-phospholipid syndrome score. Rheumatology (Oxford).

[9] Otomo K, Atsumi T, Amengual O, Fujieda Y, Kato M (2012). Efficacy of the antiphospholipid score for the diagnosis of antiphospholipid syndrome and its predictive value for thrombotic events. Arthritis and Rheumatism.

[10] Sciascia S, Sanna G, Murru V, Roccatello D, Khamashta MA (2015). The global anti-phospholipid syndrome score in primary APS. Rheumatology.

[11] Barilaro G, Esteves A, Della Rocca C, Perez-Isidro A, Araujo O (2023). Predictive value of the Adjusted Global Anti-Phospholipid Syndrome Score on clinical recurrence in APS patients: a longitudinal study. Rheumatology.

[12] Garcia L, Velloso MS, Martire MV, Savy F, Arizpe F (2020). Validation of the adjusted global antiphospholipid syndrome score in systemic lupus erythematosus patients in Argentina. Lupus.

[13] Fernandez Mosteirin N, Saez Comet L, Salvador Osuna C, Calvo Villas JM, Velilla Marco J (2017). Independent validation of the adjusted GAPSS: role of thrombotic risk assessment in the real-life setting. Lupus.

[14] Oku K, Amengual O, Bohgaki T, Horita T, Yasuda S (2015). An independent validation of the global anti-phospholipid syndrome score in a Japanese cohort of patients with autoimmune diseases. Lupus.

[15] Uludağ Ö, Bektaş M, Çene E, Sezer M, Şahinkaya Y (2021). Validation of the adjusted global antiphospholipid syndrome score in a single centre cohort of APS patients from Turkey. Journal of Thrombosis and Thrombolysis.

[16] Nascimento IS, Radin M, Gândara APR, Sciascia S, de Andrade DCO (2020). Global antiphospholipid syndrome score and anti-ß2-glycoprotein I domain I for thrombotic risk stratification in antiphospholipid syndrome: a four-year prospective study. Lupus.

[17] Radin M, Sciascia S, Erkan D, Pengo V, Tektonidou MG (2019). The adjusted global antiphospholipid syndrome score (aGAPSS) and the risk of recurrent thrombosis: results from the APS ACTION cohort. Seminars in Arthritis and Rheumatism.

[18] Petri M, Orbai AM, Alarcón GS, Gordon C, Merrill JT (2012). Derivation and validation of the Systemic Lupus International Collaborating Clinics classification criteria for systemic lupus erythematosus. Arthritis and Rheumatism.

[19] Williams B, Mancia G, Spiering W, Agabiti Rosei E, Azizi M (2018). 2018 ESC/ESH Guidelines for the management of arterial hypertension. European Heart Journal.

[20] Grundy SM, Stone NJ, Bailey AL, Beam C, Birtcher KK (2019). 2018 AHA/ACC/AACVPR/AAPA/ABC/ACPM/ADA/AGS/APhA/ASPC/NLA/PCNA Guideline on the management of blood cholesterol: A report of the American College of Cardiology/American Heart Association Task Force on Clinical Practice Guidelines. Journal of the American College of Cardiology.

[21] American Diabetes Association (2020). 2. Classification and diagnosis of diabetes: standards of medical care in diabetes-2020. Diabetes Care.

[22] Pengo V (2012). ISTH guidelines on lupus anticoagulant testing. Thrombosis Research.

[23] Pericleous C, Ferreira I, Borghi O, Pregnolato F, McDonnell T (2016). Measuring IgA anti-β2-glycoprotein I and IgG/IgA anti-domain I antibodies adds value to current serological assays for the antiphospholipid syndrome. PloS One.

[24] Sciascia S, Cosseddu D, Montaruli B, Kuzenko A, Bertero MT (2011). Risk Scale for the diagnosis of antiphospholipid syndrome. Annals of the Rheumatic Diseases.

[25] Ruffatti A, Del Ross T, Ciprian M, Nuzzo M, Rampudda M (2009). Risk factors for a first thrombotic event in antiphospholipid antibody carriers. A multicentre, retrospective follow-up study. Annals of the Rheumatic Diseases.

[26] Demir S, Li J, Magder LS, Petri M (2021). Antiphospholipid patterns predict risk of thrombosis in systemic lupus erythematosus. Rheumatology.

[27] Sciascia S, Radin M, Sanna G, Cecchi I, Roccatello D (2018). Clinical utility of the global anti-phospholipid syndrome score for risk stratification: a pooled analysis. Rheumatology.

[28] Sciascia S, Sanna G, Murru V, Roccatello D, Khamashta MA (2014). Anti-prothrombin (aPT) and anti-phosphatidylserine/prothrombin (aPS/PT) antibodies and the risk of thrombosis in the antiphospholipid syndrome. A systematic review. Thrombosis and Haemostasis.

[29] Andreoli L, Fredi M, Nalli C, Piantoni S, Reggia R (2013). Clinical significance of IgA anti-cardiolipin and IgA anti-β2glycoprotein I antibodies. Current Rheumatology Reports.

[30] De Craemer AS, Musial J, Devreese KM (2016). Role of anti-domain 1-β2 glycoprotein I antibodies in the diagnosis and risk stratification of antiphospholipid syndrome. Journal of Thrombosis and Haemostasis.

